# Contemplated Work on the Physical Condition and Diseases of the Region between the Gulf of Mexico and the Northern Lakes

**Published:** 1846-02-01

**Authors:** Daniel Drake

**Affiliations:** Medical Institute of Louisville


*Contemplated Work on the physical condition and diseases of the region between the Gulf of Mexico and the Northern Lakes, by Daniel Drake, M. D., of the Medical Institute of Louisville.*—The project of a work on the above subject was announced to the profession by Dr. Drake some years since. By the December number of the Western Journal we learn that he is now engaged in its composition, and that he looks forward to its completion at no distant day. The Doctor solicits facts bearing on the subject. We quote from his circular as follows: "Post mortem examinations, remarkable cases, and histories of epidemics of all kinds will be acceptable; in short, any well observed, and well recorded fact going to illustrate the causes, symptoms, or treatment of any of our diseases, will be gratefully received. As the morbid appearances in those who have died of our autumnal fevers have been but imperfectly set forth, he is extremely desirous of receiving cases of dissection of that kind. He is likewise anxious to obtain accounts of the typhoid fever, and the erysipelatous fever, which, for the last few years, have prevailed in various parts of the West and South."

He asks, also, for meteorological observations, to come down to the end of the year 1845; and would like to receive accurate notice of the times in the ensuing spring when the following plants unfold their leaves: peach tree, white flowering locust, dogwood, and cotton wood. "In every case a number of trees should be observed, and the aspect of the spot, in reference to the sun, recorded."

